# ATTITUDES TOWARD AGING: EDUCATIONAL INTERVENTIONS FOR MEDICAL STUDENTS

**DOI:** 10.1093/geroni/igad104.2599

**Published:** 2023-12-21

**Authors:** Bronwyn Keefe

**Affiliations:** Boston University, Boston, Massachusetts, United States

## Abstract

Ageism is stereotyping, prejudice, and discrimination based on a person’s age, and in healthcare includes the implicit biases of providers. Like many other forms of prejudice, ageism contributes to health disparities and differences in health outcomes. The implicit and explicit biases of clinicians can impact clinical practice, leading to medical decisions based on stereotypes and prejudices. There is inconclusive evidence on undergraduate and graduate medical learners’ attitudes towards aging. Some studies have found that learners hold negative associations with aging, while others have found a mix of positive and negative views toward caring for older adults. Educational interventions and intergenerational contact are two important strategies for increasing awareness and reducing ageism. Hence, it is important to discuss ageism with students and create programs and curricula to combat ageism to ensure the best care is provided to all of our patients. This session will present results from an interactive online module designed for 4th-year medical students that explores ageism and bias. Students completed the Expectations Regarding Aging Survey pre-post. Preliminary results at pre-test show that 70% believe that as they get older, they will be more forgetful and 75% believe the human body is like a car: when it’s old, it gets worn out. Healthcare professionals need to understand the existing gaps in health equity that our older adult patients experience that contribute to ageist attitudes. By drawing attention to professionals’ implicit and explicit biases towards aging throughout medical training, we are able to help older adults receive better care.

